# Blood-based test for diagnosis and functional subtyping of familial Mediterranean fever

**DOI:** 10.1136/annrheumdis-2019-216701

**Published:** 2020-04-20

**Authors:** Hanne Van Gorp, Linyan Huang, Pedro Saavedra, Marnik Vuylsteke, Tomoko Asaoka, Giusi Prencipe, Antonella Insalaco, Benson Ogunjimi, Jerold Jeyaratnam, Ilaria Cataldo, Peggy Jacques, Karim Vermaelen, Melissa Dullaers, Rik Joos, Vito Sabato, Alessandro Stella, Joost Frenkel, Fabrizio De Benedetti, Joke Dehoorne, Filomeen Haerynck, Giuseppe Calamita, Piero Portincasa, Mohamed Lamkanfi

**Affiliations:** 1 VIB Center for Inflammation Research, Zwijnaarde, Belgium; 2 Department of Internal Medicine and Paediatrics, Ghent University, Gent, Belgium; 3 School of Medical Technology, Xuzhou Medical University, Xuzhou, Jiangsu, China; 4 GNOMIXX, Statistics for Genomics, Melle, Belgium; 5 Rheumatology Unit, Bambino Gesù Children’s Hospital, Rome, Italy; 6 Department of Paediatrics, Antwerp University Hospital, Edegem, Belgium; 7 Antwerp Center for Translational Immunology and Virology (ACTIV), Vaccine & Infectious Disease Institute (VAXINFECTIO), University of Antwerp, Wilrijk, Belgium; 8 Centre for Health Economics Research & Modeling Infectious Diseases (CHERMID), Vaccine & Infectious Disease Institute (VAXINFECTIO), University of Antwerp, Wilrijk, Belgium; 9 Department of Paediatric Rheumatology, Antwerp Hospital Network, Berchem, Belgium; 10 Department of Paediatrics, University Hospital Brussel, Jette, Belgium; 11 Antwerp centre for paediatric rheumatology and auto-inflammatory diseases, Antwerp Hospital Network and Antwerp University Hospital, Antwerp, Belgium; 12 Department of Pediatric Rheumatology, University Medical Center Utrecht, Utrecht, Netherlands; 13 Department of Biosciences, Biotechnologies and Biopharmaceutics, Università degli Studi di Bari “Aldo Moro”, Bari, Italy; 14 Department of Paediatric Rheumatology, Ghent University, Gent, Belgium; 15 Tumor Immunology Laboratory, Department of Pulmonary Medicine, Ghent University Hospital, Gent, Belgium; 16 Clinical Immunology Research Lab, Centre for Primary Immunodeficiency Ghent, Ghent University Hospital, Gent, Belgium; 17 Department of Pediatric Rheumatology, Ghent University Hospital, Gent, Belgium; 18 Immunology-Allergology-Rheumatology, University of Antwerp and Antwerp University Hospital, Edegem, Belgium; 19 Division of Medical Genetics, Department of Biomedical Sciences and Human Oncology, University of Bari “Aldo Moro”, Bari, Italy; 20 Department of Paediatric Immunology and Pulmonology, Centre for Primary Immunodeficiency Ghent, Jeffrey Modell Diagnosis and Research Centre, Ghent University Hospital, Gent, Belgium; 21 Division of Internal Medicine, Department of Biomedical Sciences and Human Oncology, Clinica Medica “A Murri”, University of Bari “Aldo Moro”, Bari, Italy

**Keywords:** familial mediterranean fever, inflammation, fever syndromes

## Abstract

**Background and objective:**

Familial Mediterranean fever (FMF) is the most common monogenic autoinflammatory disease (AID) worldwide. The disease is caused by mutations in the *MEFV* gene encoding the inflammasome sensor Pyrin. Clinical diagnosis of FMF is complicated by overlap in symptoms with other diseases, and interpretation of genetic testing is confounded by the lack of a clear genotype–phenotype association for most of the 340 reported *MEFV* variants. In this study, the authors designed a functional assay and evaluated its potential in supporting FMF diagnosis.

**Methods:**

Peripheral blood mononuclear cells (PBMCs) were obtained from patients with Pyrin-associated autoinflammation with an FMF phenotype (n=43) or with autoinflammatory features not compatible with FMF (n=8), 10 asymptomatic carriers and 48 healthy donors. Sera were obtained from patients with distinct AIDs (n=10), and whole blood from a subset of patients and controls. The clinical, demographic, molecular genetic factors and other characteristics of the patient population were assessed for their impact on the diagnostic test read-out. Interleukin (IL)-1β and IL-18 levels were measured by Luminex assay.

**Results:**

The ex vivo colchicine assay may be performed on whole blood or PBMC. The functional assay robustly segregated patients with FMF from healthy controls and patients with related clinical disorders. The diagnostic test distinguished patients with classical FMF mutations (M694V, M694I, M680I, R761H) from patients with other *MEFV* mutations and variants (K695R, P369S, R202Q, E148Q) that are considered benign or of uncertain clinical significance.

**Conclusion:**

The ex vivo colchicine assay may support diagnosis of FMF and functional subtyping of Pyrin-associated autoinflammation.

**Key messages**

**What is already known about this subject?**

- Familial Mediterranean fever (FMF) is the most common monogenic autoinflammatory disease (AID), affecting an estimated 150 000 patients.
- More than 340 disease-associated variants in *MEFV*, the causal gene in FMF, have been reported.
- FMF diagnosis is primarily clinical, and further supported by review of ethnic origin, family history and genetic information.
- Diagnosis delay is common in FMF, and complicated by incomplete clinical presentation and overlap in symptoms with other periodic fever syndromes.

**What does this study add?**

- The study reports and validates a functional diagnostic test that discriminates FMF over healthy controls and related AIDs.
- The ex vivo colchicine test identifies two mechanistic subtypes of Pyrin-associated AID.

**How might this impact on clinical practice or future developments?**

- The test may help to expedite FMF diagnosis and timely initiation of colchicine therapy.

## Introduction

Monogenic autoinflammatory diseases (AIDs) are a rapidly expanding group of genetically diverse but phenotypically overlapping inflammatory disorders caused by primary dysfunction of the innate immune system.^1–3^ They are also referred to as ‘periodic fever syndromes’ because many of these diseases feature recurrent fevers and episodes of systemic or organ-specific inflammation. They can cause significant morbidity and even mortality. A clinical challenge is that efficient diagnosis is hampered by overlapping clinical features and non-specific symptoms that are shared by patients suffering from diseases with distinct aetiologies. Moreover, patients suffering from AIDs with similar underlying mechanisms, who respond to particular therapies, may present with atypical or even distinctive symptoms.^4^ While genetic testing is widely implemented for AID diagnosis, interpretation of genetic results is often challenging, which has even become more complex with the availability of next-generation sequencing allowing multiple genes to be tested simultaneously and generating an increasing number of variants, frequently of unknown significance.^5 6^ Thus, there clearly is a growing need for new or improved tools to diagnose these diseases.

With an estimated 150 000 patients, FMF is considered the most common monogenic AID worldwide, mainly affecting populations originating from the Mediterranean basin.^7^ Since its suggested use in 1972, the microtubule polymerisation inhibitor colchicine has become the gold standard for treatment in FMF, with an overall non-responder rate of only 5%–10%.^8 9^ For patients who are resistant or intolerant to colchicine, anti-IL-1 therapy is a safe and effective alternative.^10–13^ Colchicine prevents not only FMF attacks but also disease-associated complications such as amyloid A amyloidosis, a severe manifestation with poor prognosis.^9^ However, it is crucial to establish a timely and correct diagnosis of FMF before committing to daily, lifelong treatment. Current FMF diagnosis is primarily clinical, and further supported by review of ethnic origin, family history and genetic information.^7^ Robust and timely clinical diagnosis of FMF is complicated by significant overlap in symptoms and the clinical presentation of other AIDs, most of which do not respond to colchicine therapy. Additionally, interpretation of genetic testing may prove challenging with around 340 disease-associated variants in *MEFV*, the gene mutated in FMF patients, being reported in the Infevers database to date.^14–16^ Many of these variants are common in the general public, but there are also a number of rare variants of unknown pathogenicity. While in silico tools can be useful in predicting pathogenicity, care should be taken when used for clinical interpretation. Some of the most common *MEFV* mutations (M694V, M694I and M680I) are predicted as benign/non-deleterious by two such programmes, PolyPhen and SIFT, while having the most severe clinical consequences.^17 18^ In silico prediction for *MEFV* variants may be hampered by the fact that amino acids that cause human disease are often present as a wild-type allele in primates,^19^ but also by the incomplete understanding of the pathophysiological mechanisms underlying FMF. Acknowledging that the difficulties in linking genotype and phenotype in FMF are caused by an incomplete understanding of the molecular pathogenic mechanism underlying FMF, a recently reported consensus-driven pathogenicity classification was able to classify most variants in three genes causing other AID (*MVK*, *NLRP3* and *TNFRSF1A*), but almost half of the *MEFV* variants (42.4%) could not be classified or were classified as ‘variants of uncertain significance’.^5^

*MEFV* was identified as the causal gene of FMF in 1997.^14 15^ More recently, it was established that Pyrin, the protein encoded by *MEFV,* senses inactivation of RhoA GTPase, resulting in formation of an inflammasome that activates the protease caspase-1 and drives production of interleukin (IL)-1β and IL-18.^20–28^ In a prior study, we reported that in contrast to wild-type Pyrin, which requires microtubules to activate the inflammasome pathway, FMF-associated Pyrin mutants engage the inflammasome pathway independently of microtubules.^24^ Here, we report that microtubule-independent activation of the Pyrin inflammasome in the ex vivo colchicine assay is specific to FMF alleles, allowing discrimination from healthy individuals and patients suffering from Pyrin-associated AIDs that are distinct from FMF and other AIDs, including pyogenic arthritis, pyoderma gangrenosum, and acne (PAPA) and mevalonate kinase deficiency (MKD) that also have been associated with altered Pyrin inflammasome activation. Technical optimisation showed that the ex vivo colchicine assay may be performed using a small volume of human whole blood to support convenient and straightforward diagnosis of FMF. Finally, we provide an extensive validation of the ex vivo colchicine assay in a distinct population of patients suffering from FMF (n=43) and Pyrin-associated AID that is distinct from FMF (n=8). We show that the functional assay correlates with the *MEFV* genotype, and that the diagnosis of FMF almost perfectly coincides with the recently published consensus pathogenicity classification with some notable exceptions. This test thus aids in and provides further support for the pathogenicity classification of specific *MEFV* variants.

## Materials and methods

### Human whole blood

Peripheral venous blood specimens were collected from healthy individuals as well as from patients with FMF using EDTA-coated Vacutainer tubes. Whole blood was used either fresh or after overnight storage at room temperature in the dark. Whole blood was seeded, 200 μL per 96-well, and maintained in a 5% CO_2_ incubator at 37°C.

### Human PBMC isolation

Peripheral venous blood specimens were collected from healthy individuals as well as from patients suffering from FMF, PAPA or MKD. Human PBMCs were isolated from blood collected in EDTA-coated Vacutainer tubes followed by Ficoll-Hypaque density gradient centrifugation. After isolation, PBMCs were stored in liquid nitrogen for later usage. On thawing, PBMCs were allowed to recover for 1 hour at 37°C in culture medium consisting of Roswell Park Memorial Institute (RPMI) medium supplemented with 10% fetal bovine serum (FBS). Following cell viability determination, cells were seeded at a density of 2.5×10^5^ per 96-well and maintained in a 5% CO_2_ incubator at 37°C.

### Reagents and stimulation

Activation of the Pyrin inflammasome was performed by stimulating PBMCs or whole blood with *Clostridium difficile* toxin A (TcdA; 1 µg/mL; Enzo Life Sciences) alone, or with a combination of colchicine (1 µM; Sigma) and TcdA (1 µg/mL; Enzo Life Sciences). PBMC samples were incubated for 5 hours, while whole blood tests were incubated for 24 hours.

### Cytokine analysis

Human IL-1β and IL-18 cytokine levels were determined in cell culture supernatants by magnetic bead-based multiplex assay using Luminex technology (Bio-Rad). The IL-1β and IL-18 ratios were calculated by dividing the cytokine level of the combined colchicine TcdA treatment by the cytokine level of the treatment with TcdA alone. GraphPad Prism V.6.0 software was used for data analysis.

### Statistics

To evaluate the predictive accuracy of the functional assay, a receiver operating characteristic (ROC) curve was generated using GraphPad Prism V.7.01 software. The area under curve (AUC), sensitivity and specificity were calculated with the latter two being used to determine the Youden index. For the analysis of variance, a linear model of the form y=µ+*MEFV* genotype+gender+origin+age+error was fitted to the IL-1β ratio and IL-18 ratio data of the patients. The term *MEFV* genotype was constructed as a factor product of all 15 genotype variants having wild type, homozygous and heterozygous as levels. Significances of the *MEFV* genotype, gender, origin and age effects were assessed by an F test. A 2×2 table summarising the outcome of the assay and the presence or absence of a particular clinical parameter was generated, followed by a Fisher’s exact test to assess potential correlations of the clinical parameters of the patient cohort presented in online supplementary table 1 and the ex vivo colchicine assay. To examine the potential correlation between the assay and the clinical response to colchicine, a regression analysis was performed, followed by a Fisher’s unprotected least significant difference (LSD) test at the 5% significance level. For all tests, p<0.05 was considered statistically significant.

10.1136/annrheumdis-2019-216701.supp1 Supplementary data

### Ethical approval information

All patients and controls provided written informed consent for participation in the study, in accordance with ICH/GCP guidelines. Treating physicians provided information regarding the *MEFV* genotype, symptoms, treatment, age and gender for patients with FMF (see online supplementary table 1). Patients or the public were not involved in the design, or conduct, or reporting or dissemination plans of our research.

## Results

### A colchicine challenge assay to support diagnosis of FMF

We previously described the biochemical principle of a functional assay that may support diagnosis of FMF.^24^ The Pyrin inflammasome pathway is activated by toxin A from *Clostridium difficile*, resulting in the release of significant amounts of proinflammatory cytokines IL-1β and IL-18 from intoxicated monocytes and macrophages. The microtubule polymerisation inhibitor colchicine prohibits Pyrin inflammasome activation in cells expressing wild-type Pyrin. Contrastingly, cells harbouring the common and clinically severe FMF allele *MEFV* ^M694V^ engaged the Pyrin inflammasome and secreted IL-1β and IL-18 in the presence of colchicine despite inhibition of microtubule dynamics.^24^ We hypothesised that determining the ratio of the released cytokines in the presence versus absence of colchicine may provide a robust and fast functional read-out to support functional stratification and diagnosis of FMF.^24^

**Figure 1:**
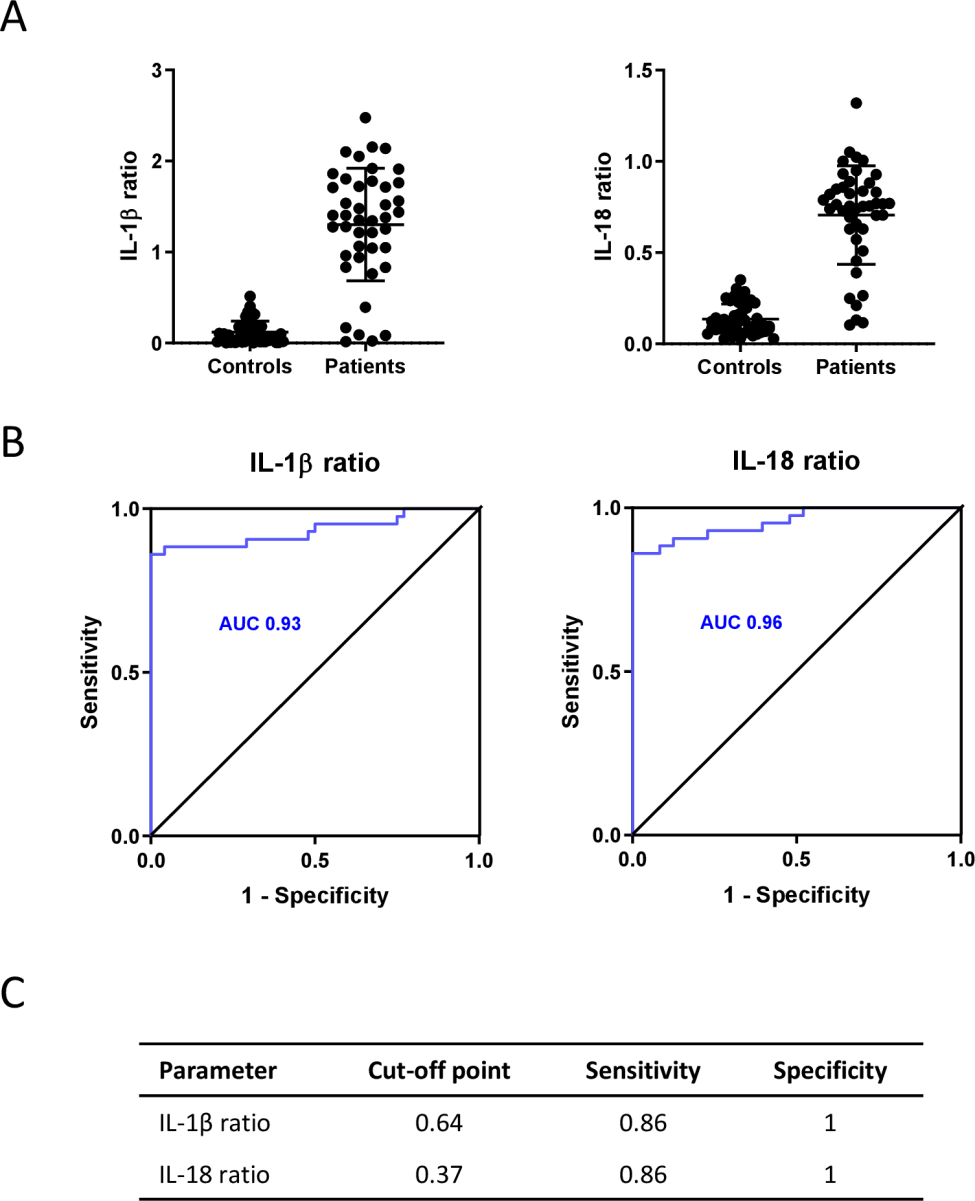
Diagnosis of familial Mediterranean fever (FMF) using a functional assay. (A) Peripheral blood mononuclear cells from healthy donors (n=48) and patients with FMF (n=43) were treated for 5 hours with either *Clostridium difficile* toxin A (TcdA) alone or with TcdA in combination with colchicine before culture supernatants were analysed for interleukin (IL)-1ß and IL-18, and the TcdA+colchicine over TcdA ratio for each cytokine was calculated. Data are combined from multiple experiments. (B) For both parameters, the receiver operating characteristic (ROC) curve was calculated, as well as the area under curve (AUC). (C) For both parameters, the Youden index was calculated to determine the most appropriate cut-off point, given the sum of sensitivity and specificity being maximum.

We performed a validation study in order to assess whether this functional test may indeed support sensitive stratification of a wider spectrum of patients with FMF differentiated according to genetic makeup, age, sex and geographical location. Additionally, we set out to compare its selectivity against healthy individuals and across a spectrum of related AID. The study group consisted of 43 patients with FMF and 8 patients with Pyrin-associated AID that was not compatible with FMF. Patients were enrolled in four hospitals located in Italy (Bari—21; Rome—8) and Belgium (Antwerp—7; Ghent—15). The median age was 20 years (2–86), and 63% patients were male. Along with the *MEFV* genotype, clinical and therapeutic characteristics of the patient group are described in online supplementary table 1. The control group consisted of 48 donors who were enrolled in three different locations (Bari—9; Rome—7; Ghent—32). Part of the blood donations for the control group were through the Red Cross, who did not pass on information regarding age and sex. Microtubule dependency was first tested for the control and FMF patient groups with both IL-1β and IL-18 ratios being used as a read-out (figure 1A). As expected,^24^ patients with FMF and healthy controls secreted IL-1β and IL-18 equally in response to *Clostridium difficile* toxin A (TcdA) alone, but contrary to patients with FMF, the cytokine ratios for healthy donors were low because of inhibition of wild-type Pyrin by colchicine (figure 1A). To evaluate the effectiveness and accuracy of the functional test, the receiver operating characteristic (ROC) curve was generated for both parameters and the area under curve (AUC) was calculated (figure 1B).^29 30^ With an AUC of 0.93 and 0.96, respectively, both parameters performed well in discriminating FMF from control samples. The Youden index was determined to establish the most appropriate cut-off value to differentiate the diseased from the non-diseased (figure 1C).^29^ This analysis resulted in respective cut-off values of 0.64 and 0.37 for the IL-1β and IL-18 ratio, maximising the specificity while maintaining a sensitivity at 0.86 for both read-outs. Inclusion in the cohort analysis of the eight patients with the prevalent *MEFV* R202Q, E148Q and P369S variants that presented with autoinflammatory features not compatible with FMF resulted in an AUC of 0.88 for both the IL-1β and IL-18 ratios, and a somewhat lower sensitivity of 0.77 for the assay (see online supplementary figure S1). In conclusion, these results suggest that the ex vivo colchicine assay can be deployed to discriminate patients with FMF from other Pyrin-associated AIDs and a control population.

### Ex vivo colchicine assay discriminates two mechanistic subtypes of FMF that correlate with pathogenicity of *MEFV* variants

**Figure 2:**
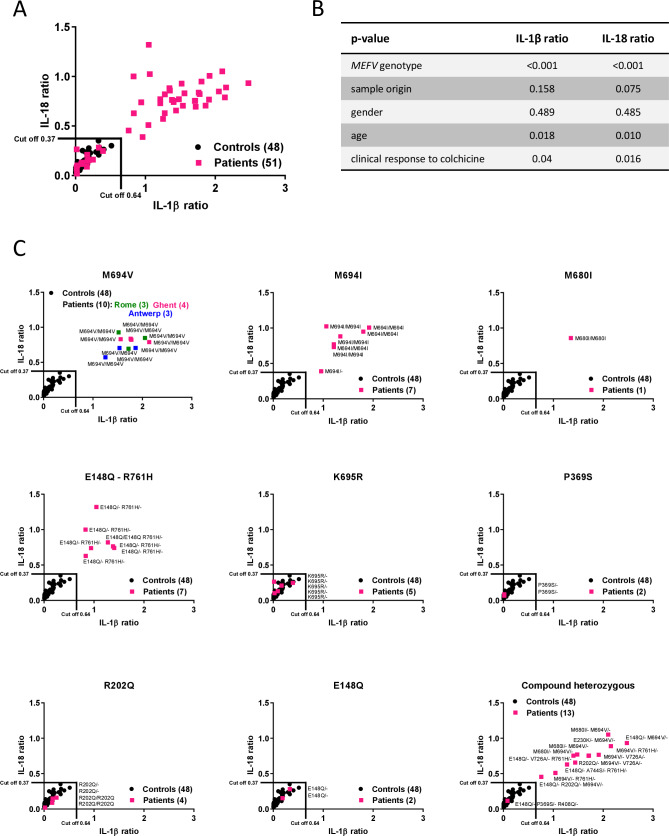
Functional stratification of patients with Pyrin-associated autoinflammatory disease correlates with *MEFV* genetic variants. (A) Combined representation of interleukin (IL)-1β and IL-18 ratios of the ex vivo colchicine assay with peripheral blood mononuclear cells from healthy donors (n=48) and the patient group composed of patients with *MEFV* gene variants that presented with either a familial Mediterranean fever (FMF) phenotype (n=43) or with autoinflammatory features not compatible with FMF (n=8). Cut-off points as determined by the Youden index are indicated. (B) Analysis of variance for the patient group represented by the p value of the F test. Regression analysis for potential correlation between the assay and clinical response to colchicine tested at 5% significance level. (C) Representation of the functional assay with patient data being separated based on *MEFV* variants.

The statistical parameters of the ROC curve demonstrate that the ex vivo colchicine assay may support the reliable identification of patients with FMF. Consistent with our previous findings,^24^ colchicine enhanced TcdA-induced IL-1β secretion in PBMCs of most patients with FMF as reflected by IL-1ß ratios>1 (figure 2A). Interestingly, early studies^31–33^ similarly reported that colchicine upregulated IL-1ß secretion and pro-IL-1ß transcript levels in lipopolysaccharide (LPS)-stimulated PBMCs, while downregulating tumour necrosis factor (TNF)-α and IL-6 levels. Further work is needed to understand the molecular mechanisms by which colchicine modulates inflammatory cytokine secretion. We next performed an analysis of variance in order to explore the epidemiological and clinical factors that correlate with the measured IL-1β and IL-18 responses in the patient group (figure 2A, B). This analysis showed that the most important contributor to the variation in the IL-1β and IL-18 ratios among patients with Pyrin-associated AID is the *MEFV* genotype, while age has a minor, but statistically significant effect. The effects of gender and the location where the samples were collected were not significant (figure 2B). The clinical parameters presented in online supplementary table 1, but amyloidosis (that did not occur in the patient cohort) were also assessed for potential correlations with the ex vivo colchicine assay by using a Fisher’s exact test. This analysis showed a lack of correlation with chest pain, abdominal pain and arthritis, with p values for these three parameters corresponding to p=0.346, p=0.467 and p=0.366, respectively. However, fever was significantly correlated with the ex vivo colchicine test (p=0.017). Notably, a regression analysis followed by a Fisher’s unprotected least significant difference test also showed a significant correlation between the ex vivo colchicine assay and the clinical response to colchicine (figure 2B). A possible explanation for this correlation is that the ex vivo colchicine assay primarily selects for patients with classical FMF mutations, the majority of whom shows a favourable clinical response to colchicine therapy (see online supplementary table 1).

Given the key role of the *MEFV* genotype, IL-1β and IL-18 ratios of patients and controls were clustered and plotted according to the *MEFV* genotype (figure 2C). Notably, this analysis showed that patients with disease-penetrant *MEFV* mutations (M694V, M694I, M680I, E148Q/R761H) are clearly separated from controls, whereas the functional response of patients with other *MEFV* variants (K695R, P369S, R202Q, E148Q) fully coincided with the controls (figure 2C). The M694V mutation located in exon 10 is considered to be the most pathogenic FMF allele causing severe disease, both in patients that are homozygous and compound heterozygous for M694V.^34–36^ The functional test described here thus objectively identifies these patients as patients with FMF, in agreement with the consensus classification (see online supplementary table 2). Patients with the M694V mutation were enrolled at three different locations (Rome, Ghent and Antwerp), all of them responding with a clear induction of Pyrin inflammasome activity in the presence of colchicine in the ex vivo colchicine test. The outcome of the test is thus independent of the location where the sample was collected. The M694I and M680I alleles also map to exon 10 and are associated with a more severe phenotype.^35^ Patients expressing these disease alleles also were clearly separated from the controls in the ex vivo colchicine assay (figure 2C), demonstrating that ex vivo colchicine testing allows identification of FMF patients with classical *MEFV* mutations. Likewise, the functional assay confirmed the pathogenic classification of variant R761H (or E148Q/R761H). Interestingly, the K695R variant—positioned adjacent to M694V in exon 10—did not cluster with the classical exon 10 FMF alleles in the functional ex vivo colchicine assay, suggesting that the functional effect of this mutation on the Pyrin inflammasome differs from the other tested disease-associated exon 10 mutations. In addition to K695R, the exons 2 and 3 *MEFV* variants in our cohort that are considered benign (R202Q) or of uncertain clinical significance (P369S and E148Q) clustered with the control population in the functional ex vivo colchicine assay (figure 2C and online supplementary table 2). All but one compound heterozygote patient in our cohort harboured at least one of the classical penetrant exon 10 FMF alleles (M680I, M694V, M694I or R761H), and were objectively reported as FMF by the ex vivo colchicine assay. The single compound heterozygous patient in our cohort that was diagnosed with variants of only uncertain clinical significance clustered with the control population in the functional assay. We also analysed PBMC from a cohort of family members of patients (n=10) to examine whether the ex vivo colchicine assay is able to identify asymptomatic carriers of FMF alleles. All nine tested carriers of penetrant exon 10 disease mutations (eight heterozygous carriers for M694V and one for M694I) clustered together with FMF patients, whereas the carrier of prevalent *MEFV* variant E148Q clustered with the healthy donor control group that lacks *MEFV* mutations (see online supplementary file 2). Together, these results highlight the clear correlation between the results of the ex vivo colchicine assay and the consensus pathogenicity classification of *MEFV* gene variants.

### Ex vivo colchicine assay distinguishes FMF from both healthy and diseased controls

**Figure 3:**
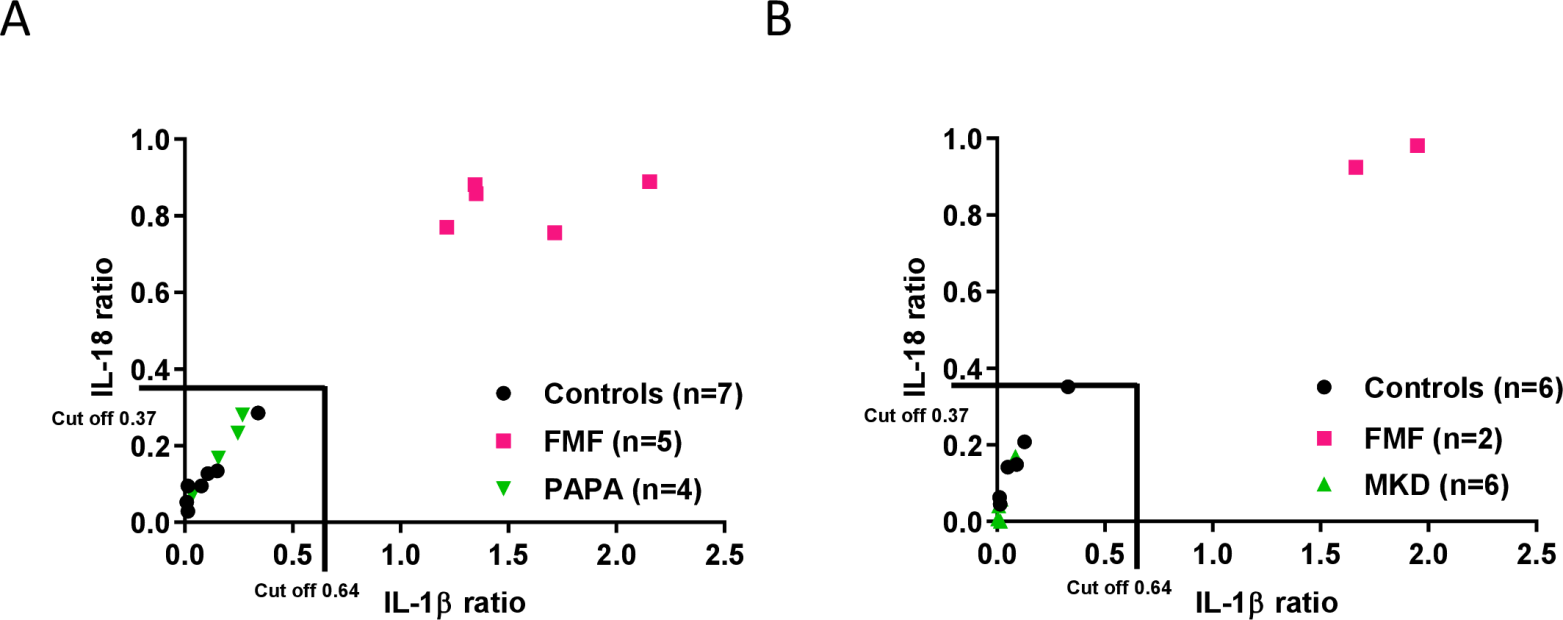
Functional stratification of familial Mediterranean fever (FMF) patients from healthy donors, pyogenic arthritis, pyoderma gangrenosum, and acne (PAPA), and mevalonate kinase deficiency (MKD) patients. Peripheral blood mononuclear cells from controls, patients with FMF, and patients with PAPA (A) or patients with MKD (B) were treated for 5 hours with either *Clostridium difficile* toxin A (TcdA) alone or TcdA in combination with colchicine before culture supernatants were analysed for interleukin (IL)-1ß and IL-18, and the TcdA+colchicine over TcdA ratio for each cytokine was calculated.

We previously demonstrated that patients afflicted with cryopyrin-associated periodic syndrome and juvenile idiopathic arthritis were classified separately from patients with FMF by the ex vivo colchicine assay.^24^ To further assess specificity of the assay, we evaluated the response of patient groups suffering from AIDs of which the pathophysiological mechanisms have been linked to deregulated activation of the Pyrin inflammasome. A first group of patients was diagnosed with PAPA syndrome, a dominantly inherited autoinflammatory disorder caused by mutations in the CD2-binding protein 1 (CD2BP1) that is predominantly mediated by granulocytes.^37^ CD2BP1 and its murine orthologue, proline-serine-threonine phosphatase interacting protein (PSTPIP1), are adaptor proteins that interact with several proteins involved in cytoskeletal organisation and inflammatory processes, including Pyrin. The mutations in *PSTPIP1* underlying PAPA syndrome trigger hyperphosphorylation and markedly increased binding to Pyrin.^38^ Consistent with the ex vivo colchicine assay being highly specific to FMF, results from the test showed that PAPA patients clustered separately from patients with FMF, thus confirming that the ex vivo colchicine assay supports reliable discrimination of patients with PAPA and FMF (figure 3A and online supplementary table 3A). Encouraged by these findings, we next examined the response of patients suffering from mevalonate kinase deficiency (MKD)/hyperimmunoglobulin D syndrome (HIDS), another inflammatory disease that has been suggested to be associated with defective geranylgeranylation of RhoA GTPase and Pyrin inflammasome activation.^20 39 40^ MKD/HIDS is caused by mutations in the *MVK* gene that target mevalonate kinase activity in the cholesterol and isoprene biosynthesis pathways.^41–43^ Although the molecular aetiology of MKD/HIDS is still debated, it is clear that reduced MVK activity leads to build-up of mevalonic acid, and a shortage of cholesterol, vitamins and other products of the isoprenoid biosynthesis pathway, which cause uncontrolled release of IL-1β through incompletely understood mechanisms. However, we found that the response of patients with PAPA in the ex vivo colchicine assay resembled that of healthy controls, with both markedly segregating from a panel of patients with FMF with classical *MEFV* mutations (figure 3B and online supplementary table 3B). Together, these results demonstrate that the ex vivo colchicine assay is able to highly specifically stratify patients with FMF from healthy donors and patients suffering from related AIDs.

### Functional FMF testing is feasible in human whole blood

**Figure 4:**
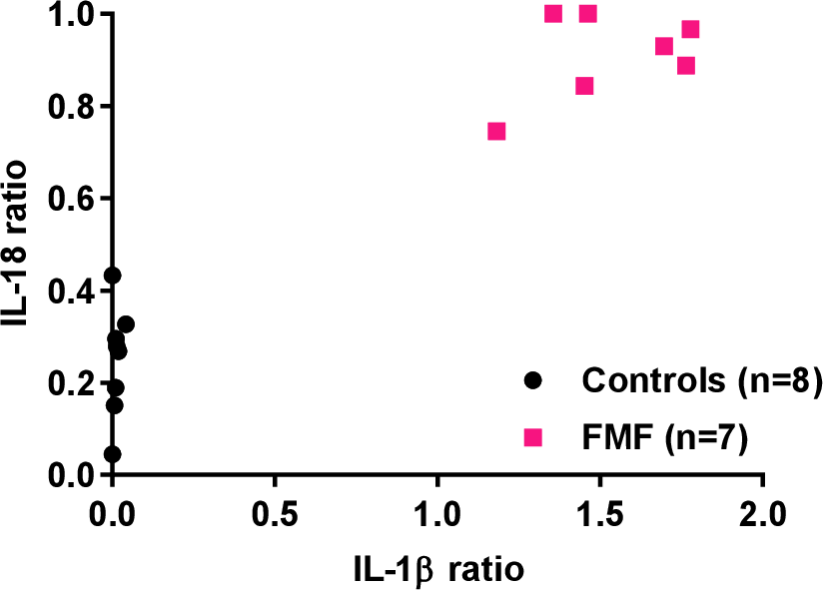
Functional familial Mediterranean fever (FMF) screening in human whole blood. Fresh, undiluted whole blood from controls (n=8) and patients with FMF(n=7) was treated for 24 hours either with *Clostridiumdifficile*toxin A (TcdA) alone or TcdA in combination with colchicine before culture supernatants were analysed for interleukin (IL)-1ß and IL-18, and the TcdA+colchicine over TcdA ratio for each cytokine was calculated. Results are combined from four independent experiments.

Although a well-established sampling method in laboratory testing of disease-specific activity for inflammatory disorders, the isolation of PBMC is a labour-consuming and time-consuming activity that requires specialised laboratory equipment that is not commonly available in clinical laboratories. Moreover, PBMC purification requires larger amounts of blood draws compared with direct whole blood analysis. In order to facilitate broad adoption of ex vivo colchicine testing in routine screening of patients suspected of FMF, we explored whether our findings with purified PBMC could be replicated in whole blood testing. Indeed, ex vivo colchicine challenge of undiluted whole blood showed a clear segregation of both the IL-1β and IL-18 ratios for FMF patients with classical *MEFV* mutations and healthy donors (figure 4 and online supplementary table 4), establishing that the functional assay can be conveniently performed on fresh blood, bypassing the need for PBMC isolation. Notably, we confirmed that the assay also works in whole blood stored overnight, although this procedure came with significantly increased background levels for IL-18, which limited the sensitivity of IL-18 ratio determination in both control and FMF samples (data not shown). Regardless, we demonstrated here that functional screening of FMF alleles based on ex vivo colchicine challenge is a technically robust procedure that may support FMF screening based on purified PBMC as well as whole blood.

## Discussion

A consensus-driven pathogenicity classification was recently proposed to support the urgent need for clear guidelines and uniform diagnosis of FMF across the world.^5^ An unforeseen outcome of this study was the large number of *MEFV* variants that were classified as ‘variants of uncertain significance’ or ‘unsolved pathogenicity’, demonstrating the urgent need for insight in the functional impact of these *MEFV* variants on Pyrin function. A functional test might shed more light on the deleterious effect of specific variants and aid in a more straightforward diagnosis of the disease as exemplified by clinical experience in XIAP (X-linked inhibitor of apoptosis) deficiency.^44^ Here, we presented and validated a robust functional assay that is able to specifically stratify patients with FMF from healthy controls, as well as from patients suffering from distinct Pyrin-associated autoinflammation and related AIDs. We demonstrated that the secretion ratios of IL-1β and IL-18 can be used together to increase the robustness of the ex vivo colchicine assay, although it remains possible to rely on a single cytokine for reading out results. When whole blood is used for testing, we noted that IL-1β outperforms IL-18, especially in samples that have been stored overnight prior initiation of the test.

We showed that the primary variable determining the outcome of the ex vivo colchicine assay is the *MEFV* genotype. While we noted that age may have a minor contribution, this may be related to the fact that patients with the most severe FMF mutations are symptomatic at a younger age. Within the FMF patient population, M680I, M694V, M694I and V726A are the most common disease-associated pathogenic mutations.^45–47^ The ex vivo colchicine assay clearly classifies patients carrying the M694V, M680I or M694I mutations as patients with FMF, thus supporting the validity of the test. Unfortunately, no conclusion could be drawn for the V726A mutation because only patients harbouring this mutation in a compound heterozygous state participated in our study.

The consensus agreement is that the E148Q variant in exon 2 is a highly prevalent *MEFV* variant of ‘uncertain clinical significance’. Results from the ex vivo colchicine assay support this assessment by showing that the functional response of patients with the E148Q variant resembles that of healthy donors expressing wild-type Pyrin, contrary to patients with disease-penetrant FMF mutations such as M694V. Moreover, colchicine was not beneficial in patients with E148Q variants included in this study, further supporting the classification of these patients as Pyrin-associated periodic fever that is distinct from FMF (see online supplementary tables S1–S2).

R202Q is another heavily debated variant. Akin to E148Q, R202Q is located in exon 2 and highly prevalent in control populations. The current consensus classifies this variant as benign.^48^ Functional evaluation in the ex vivo colchicine assay showed that the R202Q Pyrin variant responded similarly to wild-type Pyrin. Furthermore, unlike patients with classical FMF mutations, none of the R202Q-bearing patients in our study benefitted from colchicine therapy (see online supplementary table 1).

P369S is an exon 3 variant for which limited genetic and clinical data are currently available.^48^ The variant can be present by itself or as part of a complex (E148Q-P369S-R408Q)^49^ as is the case for one of the compound heterozygous patients in our patient cohort. Similar to E148Q and R202Q, P369S is highly common (2%–3% of the overall population) and is therefore to be rather considered a polymorphism. In addition, the mild phenotype and incomplete penetrance that have been reported for patients with P369S variants matches with the consensus agreement that this variant should be classified as one of ‘uncertain significance’.^45^ Notably, the ex vivo colchicine assay corroborates this conclusion by showing that the variant clearly segregated from disease-penetrant FMF mutations.

We also evaluated the R761H variant in the present study. In our patient population, this exon 10 variant was always present in combination with exon 10 mutation M694V or the exon two variant E148Q. Both the E148Q and R761H variants are usually considered low penetrance alleles, although they have been associated with FMF in patients from a recently described novel endemic area in southeastern Italy.^50^ Interestingly, patients with compound heterozygous E148Q-R761H alleles responded similarly to patients with disease-penetrant FMF mutations in the ex vivo colchicine test. Given that E148Q does not cause FMF as discussed above, we conclude that the R761H mutation renders Pyrin activation independent of microtubule dynamics, similarly to the disease-penetrant FMF mutations M680I, M694V and M694I.

The K695R allele, positioned adjacent to the most common pathogenic M694V mutation in exon 10, recently made the switch from ‘uncertain significance’ to ‘likely pathogenic’ based on the consensus agreement of an expert team.^5 7^ Our results from the ex vivo colchicine test, however, show that the functional response of this mutation clearly differs from that of classical exon 10 FMF mutations, including the adjacent M694 disease alleles, thus suggesting that Pyrin inflammasome activation in AID patients with K695R alleles may differ mechanistically from that in FMF patients with classical exon 10 mutations. Further research is required to understand how Pyrin inflammasome signalling is deregulated in patients with the K695R mutation.

Notably, we observed a significant correlation between the ex vivo colchicine assay and the clinical response to colchicine (p<0.05), possibly because the ex vivo colchicine assay primarily selects for patients with classical FMF mutations, the majority of whom shows a favourable clinical response to colchicine therapy. At first sight, this may appear paradoxical because identification of FMF alleles by the ex vivo colchicine assay is based on the inability of high colchicine concentrations (in the range of 0.1–1 μM) to inhibit inflammasome activation by FMF-associated Pyrin mutants.^24^ However, plasma concentrations of colchicine that are therapeutically effective in patients with FMF (<4 ng/mL or <0.01 µM)^51^ fail to robustly inhibit TcdA-induced secretion of IL-1ß and IL-18 from TcdA-stimulated PBMCs of healthy donors (data not shown). This suggests that colchicine likely exerts its therapeutic benefit in patients with FMF through other, yet incompletely understood mechanisms that clearly warrant further investigation,^8 51 52^ and emphasises that the functional response in the ex vivo colchicine assay should not be interpreted as a predictive marker of the clinical response to colchicine therapy.

Regardless, the ex vivo colchicine assay presented here supports straightforward functional stratification of patients with FMF. Moreover, the test may enable in-depth mechanistic studies of the many prevalent and rare *MEFV* variants and mutations to examine whether and how they impact Pyrin function to promote Pyrin-associated AID.
