# Supplementary material for: Blood-based test for diagnosis and functional subtyping of familial Mediterranean fever
Source: Ann Rheum Dis. 2020 Apr 20;79(7):960–8. doi: 10.1136/annrheumdis-2019-216701 (PMC7307214; doi:10.1136/annrheumdis-2019-216701)
Supplement: Supplementary data [file annrheumdis-2019-216701supp001.pdf]

Supplementary Data for:

A blood-based test for diagnosis and functional subtyping of Familial Mediterranean Fever

Supplementary Tables S1-S4

Supplementary Figures S1-S2

Table S1. Clinical and therapeutic characteristics of the Patient group

ID	*MEFV* variants	Symptoms					Treatment	
		Fever	Amyloidosis	Chestpain	Abdominal pain	Arthritis	Daily colchicine treatment	Patient responsive to treatment
1	M694V/M694V	yes	no	no	yes	no	yes	yes
2	M694V/M694V	yes	no	no	yes	no	yes	yes
3	M694V/M694V	yes	no	yes	yes	no	yes	partial
4	M694V/M694V	yes	no	yes	yes	yes	yes	yes
5	M694V/M694V	yes	no	yes	yes	no	yes	yes
6	M694V/M694V	yes	no	yes	yes	no	yes	yes
7	M694V/M694V	yes	no	yes	no	no	yes	yes
8	M694V/M694V	yes	no	yes	yes	yes	yes	yes
9	M694V/M694V	yes	no	yes	yes	yes	no, Canakinumab	yes
10	M694V/M694V	yes	no	yes	yes	no	yes	yes
11	M694I/M694I	yes	no	yes	yes	no	yes	no
12	M694I/M694I	no	no	no	yes	no	no, Imuran	no
13	M694I/M694I	yes	no	no	yes	yes	yes	yes
14	M694I/M694I	yes	no	no	yes	no	yes	yes
15	M694I/M694I	yes	no	no	no	yes	yes	yes
16	M694I/M694I	yes	no	no	yes	no	yes	yes
17	M694I/-	yes	no	no	no	yes	yes	no
18	M680I/M680I	yes	no	yes	yes	no	yes	yes
19	E148Q/- R761H/-	yes	no	no	no	no	yes	yes
20	E148Q/- R761H/-	yes	no	no	yes	no	yes	yes
21	E148Q/- R761H/-	yes	no	no	yes	yes	yes	yes
22	E148Q/- R761H/-	yes	no	no	yes	no	yes	yes
23	E148Q/- R761H/-	yes	no	no	yes	yes	yes	yes
24	E148Q/- R761H/-	yes	no	no	no	no	yes	yes
25	E148Q/E148Q R761H/-	yes	no	no	yes	no	yes	yes
26	K695R/-	yes	no	no	no	yes	yes	yes
27	K695R/-	no	no	no	no	no	no	
28	K695R/-	yes	no	no	yes	yes	yes	yes
29	K695R/-	yes	no	no	yes	yes	yes	yes
30	K695R/-	no	no	yes	yes	no	yes	yes
31	P369S/-	yes	no	no	yes	no	yes	yes
32	P369S/-	yes	no	yes	yes	no	yes	yes
33	R202Q/R202Q	yes	no	yes	yes	yes	yes	no
34	R202Q/R202Q	yes	no	no	yes	yes	yes	no
35	R202Q/-	yes	no	no	no	yes	yes	no
36	R202Q/-	no	no	no	yes	yes	no	
37	E148Q/-	no	no	yes	no	no	yes + ASA	partial [(a)]
38	E148Q/-	yes	no	no	yes	no	yes	no
39	E148Q/- P369S/- R408Q/-	yes	no	no	yes	no	no	
40	E230K/- M694V/-	yes	no	yes	yes	no	yes	yes
41	E148Q/- M694V/-	yes	no	no	yes	no	yes	yes
42	E148Q/- R202Q/- M694V/-	yes	no	no	yes	yes	yes	yes
43	E148Q/- V726A/- R761H/-	yes	no	no	yes	no	yes	yes
44	E148Q/- A744S/- R761H/-	yes	no	yes	no	no	yes	yes
45	R202Q/- M694V/- V726A/-	yes	no	yes	yes	yes	yes	yes
46	M680I/- M694V/-	yes	no	no	yes	no	yes	yes
47	M680I/- M694V/-	yes	no	no	yes	no	yes	yes
48	M680I/- M694V/-	yes	no	yes	yes	yes	yes	yes
49	M694V/- V726A/-	yes	no	yes	yes	yes	yes	yes
50	M694V/- R761H/-	yes	no	yes	no	no	yes	yes
51	M694V/- R761H/-	yes	no	yes	yes	yes	yes	yes

Abbreviations: ASA, acetylsalicylic acid

[(a)] tendency for flare-up of thoracic symptoms after ASA dose reduction

Table S2. Classification of Pyrin-associated autoinflammatory disease *MEFV* variants based on Infevers database and the ex vivo colchicine challenge assay

***Mefv* variant**	**Exon**	**Consensus classification Infevers database ***	**ex vivo colchicine assay**
M694V	10	Pathogenic	FMF
M694I	10	Pathogenic	FMF
M680I	10	Pathogenic	FMF
R761H	10	Likely pathogenic	FMF
K695R	10	Likely pathogenic	Non-FMF
P369S	3	Uncertain significance	Non-FMF
R202Q	2	Benign	Non-FMF
E148Q	2	Uncertain significance	Non-FMF

* Van Gijn ME, *et al.* (2018) New workflow for classification of genetic variants' pathogenicity applied to hereditary recurrent fevers by the International Study Group for Systemic Autoinflammatory Diseases (INSAID). *J Med Genet* 55(8):530-537.

Table S3. Disease-associated mutations of tested FMF, PAPA and MKD patients in Figure 3

A

**Gene**	**Patient ID**	**Disease gene mutations**
PSTPIP1	PAPA1	E250K/-
	PAPA2	E250K/-
	PAPA3	A230T/-
	PAPA4	A230T/-
MEFV	FMF1	M680I/- M694V/-
	FMF2	M680I/M680I
	FMF3	E230K/- M694V/-
	FMF4	M694I/M694I
	FMF5	M694I/M694I

B

**Gene**	**Patient ID**	**Disease gene mutations**
MVK	MKD1	A334T - A141fs
	MKD2	V377I - W62X
	MKD3	V377I - I268T
	MKD4	V377I - V377I
	MKD5	V377I - P167L
	MKD6	V377I - I268T
MEFV	FMF1	M694V/M694V
	FMF2	M694V/M694V

Table S4. Disease-associated mutations of FMF patients tested in whole blood assay in Figure 4

Gene	Patient ID	Disease gene mutations
MEFV	FMF1	M694V/M694V
	FMF2	M694V/M694V
	FMF3	M694V/M694V
	FMF4	M694I/M694I
	FMF5	M694V/- V726A/-
	FMF6	M694I/- I692del/- E148Q/-
	FMF7	M694V/M694V

Supplementary Figure Legends:

Supplementary Figure S1: Diagnosis of Familial Mediterranean Fever using a functional assay. (A) PBMCs of healthy donors (n=48) and Pyrin-associated autoinflammatory patients that presented with an FMF phenotype (n=43) or with autoinflammatory features not compatible with FMF (n=8) were treated for 5 h with either TcdA alone or with TcdA in combination with colchicine. Next the supernatant was analyzed for IL-1ß and IL-18, and for each cytokine the TcdA + colchicine over TcdA ratio was calculated. Data are combined from multiple experiments. (B) For both parameters, the receiver operating characteristic (ROC) curve was calculated, as well as the area under curve (AUC). (C) For both parameters, the Youden index was calculated to determine the most appropriate cut-off point, given the sum of sensitivity and specificity being maximum.

Supplementary Figure S2: The ex vivo colchicine assay identifies asymptomatic carriers of FMF alleles. PBMCs of asymptomatic carriers (n=10) of the depicted genotypes, related FMF patients (n=9) and healthy donors lacking *MEFV* mutations (n=48) were treated for 5 h with either TcdA alone or with TcdA in combination with colchicine before culture supernatants were analyzed for IL-1ß and IL-18, and for each cytokine the TcdA + colchicine over TcdA ratio was calculated.

A

B

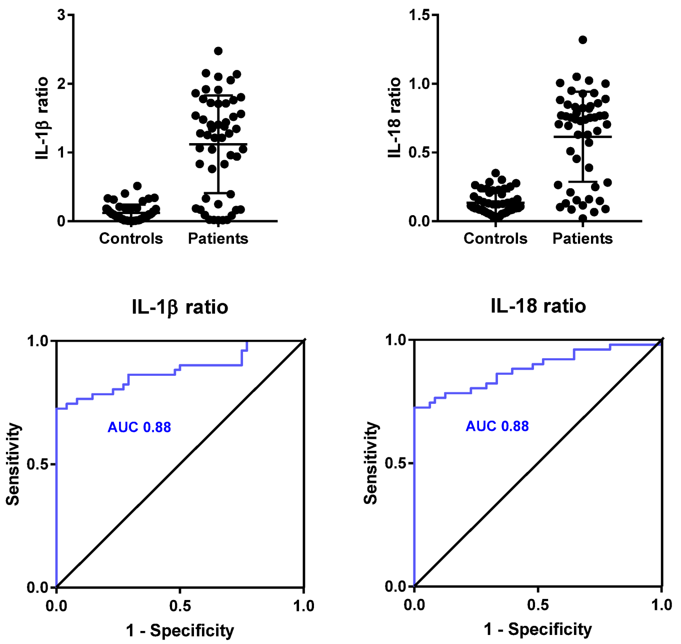

C

Parameter	Cut-off point	Sensitivity	Specificity
IL-1β ratio	0.64	0.73	1
IL-18 ratio	0.37	0.73	1

Figure S1

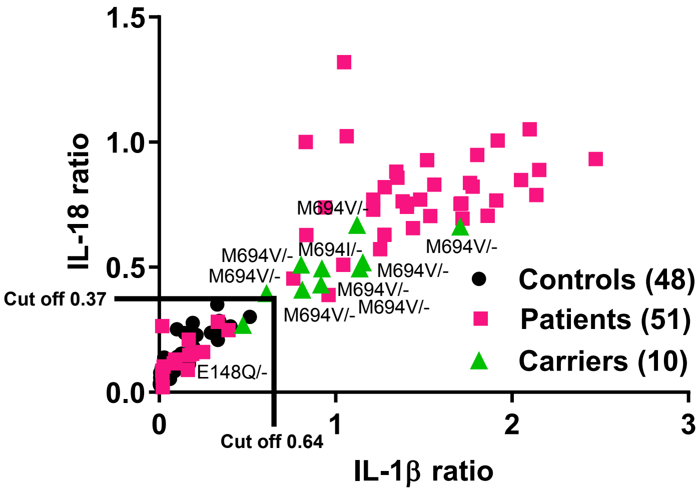

1.5
1.0
0.5
0.0
IL-18 ratio
Cut off 0.37
M694V/-
M694V/-
M694V/-
M694I/-
M694V/-
M694V/-
M694V/-
M694V/-
M694V/-
E148Q/-
0
1
2
3
Cut off 0.64
IL-1β ratio
Controls (48)
Patients (51)
Carriers (10)

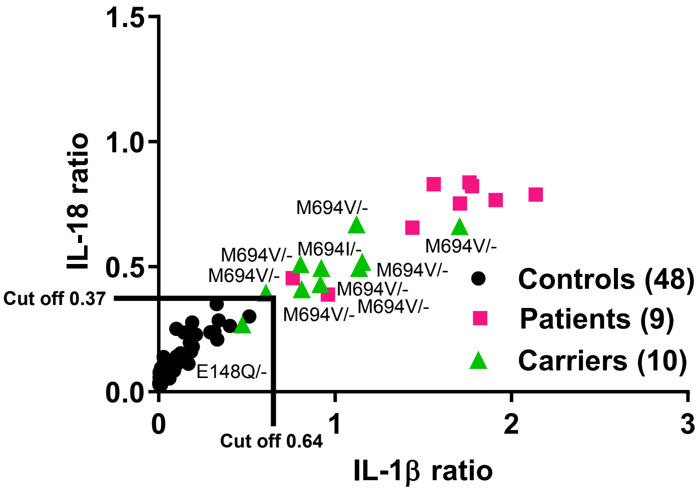

1.5
1.0
0.5
0.0
IL-18 ratio
Cut off 0.37
M694V/-
M694V/-
M694V/-
M694I/-
M694V/-
M694V/-
M694V/-
M694V/-
M694V/-
E148Q/-
0
1
2
3
Cut off 0.64
IL-1β ratio
Controls (48)
Patients (9)
Carriers (10)

Figure S2
